# A Poly(cobaloxime)/Carbon Nanotube Electrode: Freestanding Buckypaper with Polymer‐Enhanced H_2_‐Evolution Performance

**DOI:** 10.1002/anie.201511378

**Published:** 2016-02-18

**Authors:** Bertrand Reuillard, Julien Warnan, Jane J. Leung, David W. Wakerley, Erwin Reisner

**Affiliations:** ^1^ Christian Doppler Laboratory for Sustainable SynGas ChemistryDepartment of ChemistryUniversity of CambridgeLensfield RoadCB2 1EWCambridgeUK

**Keywords:** buckypaper, cobaloximes, electrocatalysis, dihydrogen evolution, polymers

## Abstract

A freestanding H_2_‐evolution electrode consisting of a copolymer‐embedded cobaloxime integrated into a multiwall carbon nanotube matrix by π–π interactions is reported. This electrode is straightforward to assemble and displays high activity towards hydrogen evolution in near‐neutral pH solution under inert and aerobic conditions, with a cobalt‐based turnover number (TON_Co_) of up to 420. An analogous electrode with a monomeric cobaloxime showed less activity with a TON_Co_ of only 80. These results suggest that, in addition to the high surface area of the porous network of the buckypaper, the polymeric scaffold provides a stabilizing environment to the catalyst, leading to further enhancement in catalytic performance. We have therefore established that the use of a multifunctional copolymeric architecture is a viable strategy to enhance the performance of molecular electrocatalysts.

Efficient, inexpensive, and scalable H_2_ production by water electrolysis driven by renewable energy sources remains a major goal towards realizing a sustainable energy cycle.^1^, ^2^, ^3^ The development of low‐cost catalysts, such as those based on the earth‐abundant 3d transition metals Co, Ni, and Fe, offers a promising route to compete with the performance of the current benchmark catalysts, including expensive platinum and fragile H_2_‐producing enzymes known as hydrogenases.^4^, ^5^, ^6^, ^7^, ^8^

Many molecular cobalt‐containing H_2_ evolution catalysts have been reported,^9^ and cobaloximes and their derivatives are among the most widely studied.^10^, ^11^, ^12^, ^13^ The advantages of cobaloximes are their facile synthesis and the tunability of their catalytic properties by modifying the substituents on the equatorial and/or axial ligands.^13^, ^14^, ^15^, ^16^ Moreover, they exhibit high proton‐reduction activity at a relatively low overpotential^17^ and can operate in aqueous solutions^18^, ^19^ even under aerobic conditions.^20^, ^21^ Recently, versatile strategies were reported for their immobilization onto metal‐oxide^13^, ^22^ and carbon‐based^23^, ^24^, ^25^ surfaces for applications in electro‐ and photoelectrocatalytic H_2_ production. Despite this progress, a major drawback and limitation in their development is their poor stability, which is mainly due to degradation of the equatorial ligand or decoordination of the axial ligand under catalytic conditions.^13^, ^26^

Carbon nanotubes (CNTs) are widely used as an electrode material because of their ability to increase the surface loading of catalytic species whilst maintaining excellent conductivity.^27^, ^28^ They can also be covalently and noncovalently functionalized with ease by using a wide range of methods.^29^, ^30^, ^31^ Strong π–π interactions between pyrene units and the CNT sidewalls have become a successful approach to anchor molecular catalysts,^32^, ^33^, ^34^, ^35^ redox probes,^36^ and proteins.^37^, ^38^ These noncovalent interactions between hydrophobic moieties and CNT sidewalls have been studied to improve the formation of robust, freestanding CNT electrodes particularly suited for electrocatalytic applications.^39^ Such CNT films are known as buckypapers (BPs) and can be easily obtained by vacuum filtration of CNT dispersions to produce porous networks of nanotubes with unique electrical conductivity, gas permeability, and mechanical properties.^39^, ^40^ The mechanical stability and high surface area of BPs have recently been shown to enhance the catalytic properties of bioelectrodes (high protein loading, efficient electronic communication, improved stability) for sensors and enzymatic biofuel cells.^41^, ^42^ The freestanding nature of these BPs precludes the need for an additional conductor or supporting scaffold, which is a considerable advantage when large‐scale electrodes are required.

The immobilization of molecular catalysts onto electrodes with high stability and performance remains a major challenge in electrocatalysis.^4^ Herein, we report the combination of multiwall carbon nanotubes (MWCNTs) with a pyrene‐bearing cobaloxime copolymer (pPyCo) to produce a functional BP for catalytic hydrogen evolution (Figure 1 a, b) and compare it with an analogous monomeric pyrene‐functionalized cobaloxime (PyCo). Both catalysts employed the same 4‐*N*‐amidylpyridine moiety as a direct or indirect linker between the catalyst and a pyrene anchoring group in the monomeric and polymeric architectures, respectively.

**Figure 1:**
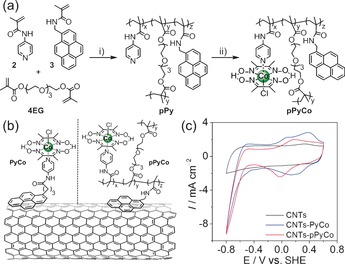
a) i) Synthetic route to pPyCo: 2,2′‐azobisisobutyronitrile (AIBN), THF, 70 °C, 16 h, 88 %. ii) [CoCl_2_(dmgH)(dmgH_2_)], Et_3_N, MeOH/CHCl_3_ (1:4), 45 °C, 4 h, 69 %. b) Schematic representation of PyCo and pPyCo catalysts attached on the MWCNT sidewalls. c) CV scans of the bare GC/MWCNTs (black), GC/MWCNTs modified with PyCo (blue) and with pPyCo (red curve) in phosphate electrolyte solution (0.1 m, pH 6.5; *ν*=100 mV s^−1^) under inert atmosphere and at room temperature.

Although only a few reports on the use of polymers in H_2_‐evolution catalysis are available,^43^, ^44^, ^45^ it is well‐established that polymers, and especially copolymers, can provide several advantages over molecular species as they can incorporate complementary properties, such as ionic conductivity,^46^ robust immobilization, and a protecting matrix, therefore resulting in a beneficial environment for the catalysts.^47^, ^48^, ^49^ By embedding a benchmark catalyst in a rationally designed copolymer, we aim to demonstrate, as a proof of concept, that tuning the electrocatalyst environment (outer coordination sphere), instead of the active catalytic center (first coordination sphere), could be a promising strategy to improve the performance of a H_2_ evolution molecular catalyst. This is, to the best of our knowledge, the first reported attempt at a rationally designed copolymer for H_2_ evolution that mimics the function of the protein matrix surrounding an enzyme's active site.

Molecular PyCo (Figure 1 b) was prepared by coordinating 4‐(pyren‐1‐yl)‐*N*‐(pyridin‐4‐yl)butanamide (**1**, see the Supporting Information) to [Co^III^Cl_2_(dmgH)(dmgH_2_)] (dmgH_2_=dimethylglyoxime). The polymeric pPy intermediate was obtained from pyridine‐based (**2**) and pyrene‐based (**3**) methacrylate monomers, which were prepared and combined together (in stoichiometric amounts) with tetraethylene glycol dimethacrylate (4EG) through free‐radical polymerization (Figure 1 a). This polymerization is a versatile metal‐free process, tolerant to a wide range of chemical functionalities that allows simple tailoring of the polymer's properties by the incorporation of specific functional groups. After the polymerization, the cobaloxime was finally introduced through the binding of the free pyridine units to the [Co^III^Cl_2_(dmgH)(dmgH_2_)] precursor. Compound pPyCo was found to be soluble in *N*,*N*‐dimethylformamide (DMF) and dimethyl sulfoxide (DMSO) despite its cross‐linked nature, probably because of a relatively low average molecular weight (*M*_w_≈10.7 kD; *Đ*_M_≈1.29). All synthetic procedures and characterization (^1^H and ^13^C NMR, FTIR spectra, high‐resolution mass spectra, elemental analysis and gel permeation chromatography) are available in the Supporting Information.

Each monomer unit in pPyCo was chosen to afford specific properties to the resultant multifunctional copolymer. The pyrene unit provides a hydrophobic anchor for binding the MWCNT sidewalls, the pyridine confers a receptor to coordinate [Co^III^Cl_2_(dmgH)(dmgH_2_)], and the 4EG improves the polymer's solubility in polar solvents. The natural hydrophilicity of 4EG also affords a more proton‐rich environment in the vicinity of the catalyst and could reinforce the stability of the global architecture because of a cross‐linked matrix. The ratio of monomers inside the polymer was estimated by ^1^H NMR and elemental analysis (H, C, N, Cl), which showed a pyrene/pyridine/4EG composition of approximately 1:1:1.3.

Initially, glassy carbon/multiwall carbon nanotube (GC/MWCNT) electrodes were modified with PyCo and pPyCo in order to fully characterize the electrochemical behavior of both the single molecule and the polymer at the surface of the MWCNTs. These GC/MWCNT electrodes were obtained by drop‐coating 20 μL of a 5 mg mL^−1^ dispersion of MWCNTs in *N*‐methylpyrrolidone onto the GC disk electrode (Ø=3 mm). After drying under vacuum, a 5 μm thick homogeneous MWCNT film was obtained as reported previously.^35^ The GC/MWCNT electrodes were then incubated for 30 min in a 2.5 mm solution of PyCo (GC/MWCNT‐PyCo) or pPyCo (GC/MWCNT‐pPyCo) in DMF and rinsed with DMF and H_2_O before running cyclic voltammetry (CV) experiments in an aqueous pH 6.5 electrolyte solution (Figure 1 c). The GC/MWCNT‐PyCo electrodes displayed a reversible redox wave at *E*_1/2_=0.24 V versus the standard hydrogen electrode (SHE), whereas a reversible process was observed at *E*_1/2_=0.08 V versus SHE with GC/MWCNT‐pPyCo. These redox processes are attributed to the cobaloximes’ reversible Co^III^/Co^II^ redox couple.^16^ The small shift in redox potential between the monomer and polymer is presumably due to a change in the chemical environment surrounding the cobalt center upon incorporation into a polymer chain. The small redox waves observed in the case of GC/MWCNT‐PyCo at *E*_red_=0.13 V and *E*_ox_=0.08 V may be the result of different adsorption interactions of the PyCo generating varying chemical environments on the GC/MWCNT surface. In contrast, the single redox process observed with GC/MWCNT‐pPyCo results most likely from a more homogenous environment around the cobalt catalysts, provided by the polymeric matrix.

Immobilization was further confirmed by the linear dependence of the Co^III^/Co^II^ redox couple current densities with scan rate for both PyCo and pPyCo (Figure S1). The Δ*E* value for the Co^III^/Co^II^ redox couple also increases with scan rate. According to the Laviron equation for interfacial electron transfer with adsorbed redox systems, Δ*E* observed for Co^III^/Co^II^ corresponds to a heterogeneous electron transfer rate (*k*_s_) of 0.23±0.05 cm s^−1^ and 0.32±0.08 cm s^−1^ for PyCo and pPyCo, respectively.^50^ The relatively close *k*_s_ values are good confirmation that the polymer structure of pPyCo does not act as an insulating layer and instead retains electron‐transfer properties comparable to PyCo within the MWCNT electrode.

A strong wave with a cathodic onset potential of *E*_onset_=−0.48 V versus SHE is observed for both GC/MWCNT‐PyCo and GC/MWCNT‐pPyCo as a result of catalytic reduction of protons to H_2_ by the reduced Co^I^ species of the cobaloxime.^16^ This value corresponds to a small overpotential for proton reduction (*η*≈100 mV), which is in agreement with previous studies for [CoCl(dmgH)_2_(pyridine)]‐type catalysts.^17^ Therefore, the observed redox and catalytic waves on these modified electrodes demonstrate that both cobaloxime‐based compounds retain their integrity and activity once immobilized on the MWCNT surface. From the integration of the Co^II^ to Co^III^ oxidation wave in the CV scans, the charge was calculated and the amount of cobalt catalyst immobilized was estimated to be approximately 33 nmol cm^−2^ and 25 nmol cm^−2^ for PyCo and pPyCo, respectively. These surface coverages are higher than for previously reported covalently immobilized cobaloximes on CNTs^24^ and are comparable to metal oxide sensitization,^13^ thus underlining the potential of soft and straightforward supramolecular functionalization methods of CNT to assemble molecular‐based electrodes.

Subsequently, MWCNT BPs were prepared in order to build electrocatalytic freestanding electrodes and to increase the specific surface area, thereby allowing the immobilization of a high amount of catalytic centers without any need for an additional electrode support material. BP‐PyCo and BP‐pPyCo were obtained by filtration of a dispersion of MWCNTs and the corresponding molecular catalysts (1:1 ratio, w/w) in a DMF solution onto a polytetrafluoroethylene (PTFE) membrane, giving reproducible and stable MWCNT disks (Figure 2 a; a detailed experimental procedure is described in the Supporting Information). A bare BP and BP‐pPy were also produced by the same procedure for comparison with the [Co]‐functionalized BPs.

**Figure 2:**
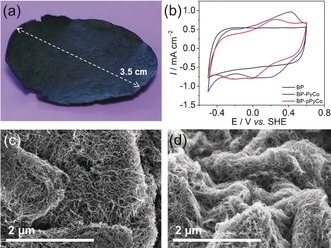
a) Photograph of a BP electrode (Ø=3.5 cm). b) CV scans of the different BP electrodes: bare BP (black), BP‐PyCo (blue), and BP‐pPyCo (red curve) recorded in phosphate electrolyte solution (0.1 m, pH 6.5; *ν*=5 mV s^−1^) under inert atmosphere and at room temperature. SEM images of c) BP‐PyCo and d) BP‐pPyCo.

Attenuated total reflectance Fourier‐transform infrared (ATR‐FTIR) spectroscopy of the functionalized BPs shows comparable features compared to those of PyCo and pPyCo (Figure S2). In particular, characteristic bands are observed at the cobaloxime's $\tilde{\nu }$ (NO^−^) stretching frequency at around 1240 cm^−1^ (Figure S2a), overlapping with the vibrational mode of the MWCNTs in the case of the BPs (Figure S2b).^45^ The polymer binding is also observable through the intense $\tilde{\nu }$ (C=O) vibrational mode at 1737 cm^−1^ that arises from the ester groups.

X‐ray photoelectron spectroscopy (XPS) experiments show characteristic cobalt, oxygen, and nitrogen binding energy peaks (Figure S3). In the Co_2p_ region, two broad signals corresponding to 2p_1/2_ and 2p_3/2_ core levels were observed at 796.8 and 781.8 eV, respectively, after BP functionalization (similar to values previously reported for a cobaloxime).^24^ Similarly, peaks in the N_1s_ and stronger O_1s_ core level regions at 400.5 and 532.7 eV, respectively, arise from the ligands and copolymer components, further indicating the successful immobilization of both PyCo and pPyCo.

Thermogravimetric analysis (TGA; under N_2_, gradient 10 °C min^−1^) showed the presence of degradable materials on the surface of the functionalized BPs, whereas no significant loss of weight is detected on the bare BP (Figure S4). Weight loss of the functionalized BP‐PyCo and BP‐pPyCo stabilized at 500 °C at approximately 6 % and 23 %, respectively, corresponding to the degradation of the organic materials physisorbed on the CNT surface. These results suggest a higher loading of material with the polymer pPyCo than with PyCo.

Finally, scanning electron microscopy (SEM) of BP‐PyCo and BP‐pPyCo showed the high surface area of the porous CNT network of the BP electrodes (Figures 2 c, d and S5). The presence of the polymer seems to limit dense packing of the CNTs, instead encouraging a rougher and more porous morphology than its monomeric counterpart.

The functionalized BPs were employed as freestanding electrodes (geometric surface=1 cm^2^) and connected directly to a potentiostat in an aqueous electrolyte solution (phosphate electrolyte solution, 0.1 m, pH 6.5). CV scans of BP‐PyCo and BP‐pPyCo exhibit a reversible Co^III^/Co^II^ redox wave at *E*_1/2_=0.18 V and *E*_1/2_=0.10 V versus SHE, respectively (Figure 2 b and S6). Interestingly, the scale‐up process to the BP electrode did not induce strong changes in the behavior of PyCo and pPyCo, as they retained environment‐controlled redox activities similar to those seen on the GC electrodes (see above). The onset potential of the catalytic proton reduction wave is also observed at around *E*_onset_≈−0.48 V versus SHE for both functionalized BPs because of the presence of the same active species within the freestanding electrode (Figure S6). Integration of the Co^II^ to Co^III^ oxidation wave allowed an estimation of the amount of immobilized Co of approximately 200 nmol cm^−2^ and 150 nmol cm^−2^ for BP‐PyCo and BP‐pPyCo, respectively. This high Co loading corresponds to an almost tenfold increase compared to the GC/MWCNT electrodes (see above) and highlights the ability of the freestanding BP electrode preparation method to load high amounts of the molecular catalysts throughout the three‐dimensional film. The slightly lower loading of Co in BP‐pPyCo suggests that more of the degradable material in the TGA measurements stems from the polymeric chain. Ultimately, this BP approach can be seen as a versatile platform from which further tuning of catalyst loading and electrode size is possible.

The freestanding BPs were evaluated for H_2_ evolution by controlled‐potential electrolysis (CPE) with an applied potential (*E*_appl_) of −0.7 V versus SHE. CPE was carried out in aqueous phosphate electrolyte solution at pH 6.5 in an airtight two‐compartment electrochemical cell under an inert atmosphere (N_2_/CH_4_, 98:2; Figure S7a). In a control experiment, the current for the bare BP electrode decreases within a few seconds to 0.10 mA cm^−2^ and remains stable at this lower current density for 24 h. BP‐PyCo exhibits higher current densities (>0.5 mA cm^−2^) for the first 15 min of electrolysis before slowly decreasing over 8 h and stabilizing at 0.12 mA cm^−2^. BP‐pPyCo displays a substantially improved performance with higher and more stable current densities of 0.34 mA cm^−2^ for 5 h, before slowly decaying over 10 h to finally stabilize at 0.12 mA cm^−2^. Knowledge of the amount of Co on the electrode, along with H_2_ produced and charge generated during CPE allowed us to calculate turnover numbers (TONs), turnover frequencies (TOFs), and faradaic yields for the functionalized BP electrodes (background proton reduction activity on cobaloxime‐free BP subtracted).

Figure 3 a depicts H_2_ production over time during CPE of BP‐PyCo and BP‐pPyCo under inert atmosphere and under air. Experiments were performed under air to assess the catalytic activity and efficiency under demanding aerobic conditions as some O_2_ tolerance of a H_2_‐generating cathode is required in water‐splitting applications.^51^ Under an N_2_ atmosphere, BP‐PyCo exhibits a linear H_2_ evolution rate for the first 3 h before slowing down and becoming almost inactive after 8 h. The Co‐based TON (TON_Co_) reaches a maximum of 80 mol H_2_ (mol Co)^−1^ (Figure 3 b), comparable to previously reported values for cobaloxime compounds in solution.^12^, ^20^ TOF_Co_ for BP‐PyCo reaches a maximum of 32 h^−1^ (Figure S7b) and decreases quickly, thus suggesting a fairly rapid deactivation of the catalyst during CPE, most probably arising from decoordination of the cobaloxime from the labile pyridine ligand during the catalytic cycle,^52^ or degradation of the catalyst.^26^

**Figure 3:**
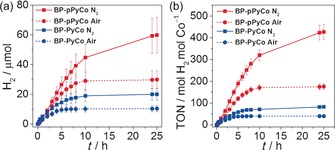
a) Electrocatalytic H_2_ production and b) TON_Co_ produced, during CPE of BP‐PyCo (blue) and BP‐pPyCo (red curves) at *E*_appl_=−0.7 V versus SHE in phosphate electrolyte solution (0.1 m, pH 6.5), under N_2_ (solid) and air (dotted curves) at room temperature.

The rate of H_2_ production remains linear for 8 h in the case of CPE with BP‐pPyCo before decaying and stabilizing at a maximal value of 60 μmol H_2_ after 24 h, which corresponds to a TON_Co_ of 420 (Figure 3). The TOF_Co_ with BP‐pPyCo remains stable at approximately 38 h^−1^ for several hours (Figure S7b), which demonstrates an improved activity for the pPyCo‐ over the PyCo‐modified BPs. The faradaic yields calculated from the charge passed during the CPE measurements confirm BP‐pPyCo's superior catalytic activity compared to BP‐PyCo with faradaic yields up to 90 % and 70 %, respectively (Figure S8). This substantially higher activity and efficiency of the polymer‐based cobaloxime demonstrates the advantages of the multifunctional polymer in potentially conferring the composite with a higher proton affinity and limiting the decomposition of the catalyst. Concurrently, the greater stability could be a result of the steric hindrance provided by the polymeric matrix limiting the loss of individual metal centers from the labile pyridines and degradation of the cobaloxime. XPS experiments conducted after electrolysis showed that the Co_2p_ signal maxima shifted from 796.8 and 781.8 eV to 798.2 and 782.2 eV concomitantly with the appearance of an overlapping shoulder (Figure S9). Nevertheless, no metallic cobalt (778.0 eV) was detected. As the catalytic activity ceased, the modification of the Co environment is most likely a consequence of the cobaloxime degrading into an inactive Co species.

A respectable level of hydrogen evolution activity is maintained under air, with 30 μmol of H_2_ (TON_Co_=180) evolved in the case of BP‐pPyCo and 10 μmol (TON_Co_=40) for BP‐PyCo (Figure 3 a). These values correspond to 45–50 % of total H_2_ produced under an inert atmosphere (Figure 3 b). As a result, faradaic yields remain significant under air with 45 % for pPyCo and 30 % for PyCo (Figure S9). Interestingly, these faradaic yields are higher than the values reported for similar molecular catalysts under air in the absence of a CNT scaffold,^21^ and highlights the protecting effect of our hybrid composite material. The loss in faradaic yield under air as compared to that under N_2_ is due to the competing reduction of oxygen by the cobaloxime.^25^, ^51^ However, the CNTs themselves can also reduce O_2_ and therefore act as a shield protecting the catalyst against the parasitic reduction of O_2_ instead of protons, enhancing the molecular catalyst's efficiency under aerobic conditions.^53^ Thus, defensive (reduction of O_2_ by the electrode scaffold protecting the catalyst from exposure to O_2_) and offensive (the cobaloxime's own reduction of O_2_ without being damaged) strategies are used to build a relatively air‐tolerant cathode for H_2_ evolution.^51^

In summary, we have described the facile association of a three‐dimensional freestanding MWCNT and polymeric catalyst for H_2_ evolution. The multifunctional poly(cobaloxime)/CNT composite enables a high loading of the molecular catalyst, catalyst stabilization and a protecting effect against O_2_ damage. As a result, the polymeric hybrid material displays a catalytic activity four times higher and twice as long compared to its monomeric counterpart. Much like for an enzyme where the active site is embedded in a protein matrix, the polymer can be considered as an activating and protecting environment for the cobaloxime catalyst core. We demonstrate the possibility to improve the catalytic properties of a Co‐based catalyst by using a simple and tailor‐made polymer backbone. The latter confers desirable characteristics to the composite and therefore prolongs the molecular catalyst's stability on the electrode scaffold without the need for Nafion^®^ or precious metals. The strategy of integrating active molecular catalysts in rationally designed polymers combined with the straightforward and scalable concept of MWCNT matrices could enable new applications for molecular catalysts in the field of renewable fuel and chemical synthesis.

## Supporting information

As a service to our authors and readers, this journal provides supporting information supplied by the authors. Such materials are peer reviewed and may be re‐organized for online delivery, but are not copy‐edited or typeset. Technical support issues arising from supporting information (other than missing files) should be addressed to the authors.

Supplementary
