# Supplementary material for: A Poly(cobaloxime)/Carbon Nanotube Electrode: Freestanding Buckypaper with Polymer‐Enhanced H2‐Evolution Performance
Source: Angew Chem Int Ed Engl. 2016 Feb 18;55(12):3952–7. doi: 10.1002/anie.201511378 (PMC4794774; doi:10.1002/anie.201511378)
Supplement: Supplementary file 1 — Supplementary [file ANIE-55-3952-s001.pdf]

Angewandte Chemie
Eine Zeitschrift der Gesellschaft Deutscher Chemiker

Supporting Information

A Poly(cobaloxime)/Carbon Nanotube Electrode: Freestanding Buckypaper with Polymer-Enhanced H_2-Evolution Performance

*Bertrand Reuillard[+], Julien Warnan[+], Jane J. Leung, David W. Wakerley, and Erwin Reisner**

anie_201511378_sm_miscellaneous_information.pdf

Contents

Experimental Section Page S2
Supporting Figures Page S7

Experimental Section

General considerations. Column chromatography was carried out with silica gel 60 (0.040-0.063 mm mesh) from Material Harvest. Tetrahydrofuran (THF), triethylamine (Et_3N) and dichloromethane (DCM) were distilled on sodium (THF) or calcium hydride (Et_3N and DCM) before use. 2,2′-azobis(2-methylpropionitrile) (AIBN) was commercially available and recrystallized prior to use from methanol (T < 40 °C) and shielded from ambient light (CAUTION: violent self-decomposition or self-ignition may be triggered by heat). Tetraethylene glycol dimethacrylate was quickly filtered over basic alumina before use to remove stabilizing agents. [$CoCl_2$(dmgH)($dmgH_2$)] ($dmgH_2$ = dimethylglyoxime) was synthesized following a previously published procedure [W. C. Trogler, R. C. Stewart, L. A. Epps, L. G. Marzilli, *Inorg. Chem*. **1974**, *13*, 1564–1570]. Multiwall carbon nanotubes (thin and short, 755117 Aldrich, purity 95%) were purchased from Sigma-Aldrich. Other chemicals and solvents were purchased from commercial suppliers and used as received.

Characterization. ^1H and ^{13}C NMR spectra were recorded on a Bruker DPX-400 MHz or a Bruker 500 MHz DCH Cryoprobe Spectrometer at room temperature. Chemical shifts are given in ppm and coupling constants in Hz. Chemical shifts for ^1H NMR spectra are referenced relative to residual protium in the deuterated solvent ($CHCl_3$: δ_H = 7.26 ppm for ^1H NMR and δ_C = 77.16 ppm for ^{13}C NMR; DMSO: δ_H = 2.50 ppm for ^1H NMR and δ_C = 39.52 ppm for ^{13}C NMR). High resolution-mass spectra were recorded using a ThermoScientific Orbitrap Classic mass spectrometer. Attenuated total reflectance FT-IR spectra of the compounds or crushed buckypapers were recorded on a Nicolet iS50 spectrometer. Elemental analysis was carried out by the Microanalysis Service of the Department of Chemistry, University of Cambridge, using a Perkin-Elmer 240 Elemental Analyser. SEM images were recorded on an FEI Magellan™ XHR SEM instrument at 2 kV acceleration voltage. X-ray photoelectron spectroscopy (XPS) measurements were carried out by the National EPSRC XPS users' service (NEXUS) at Newcastle University. Thermogravimetric Analysis (TGA) was conducted under nitrogen from 40 °C to 500 °C with a 10 °C min^{-1} gradient on a METTLER TOLEDO TGA/DSC 1 Star System. The weight average molecular weight (M_w), number average molecular weight (M_n) and dispersity ($Đ_M$) were determined by gel permeation chromatography (GPC), using a 0.7 mL min^{-1} flow rate, in *N*,*N*-dimethylformamide + LiBr (0.1 M), at 65 °C on a "Jordi xStream H_2O 10^3 Å" column connected to a Shimadzu SPD-M20A UV-vis calibrated to poly(methylmethacrylate) standards. Samples were filtered over 0.45 mm nylon filters before injection.

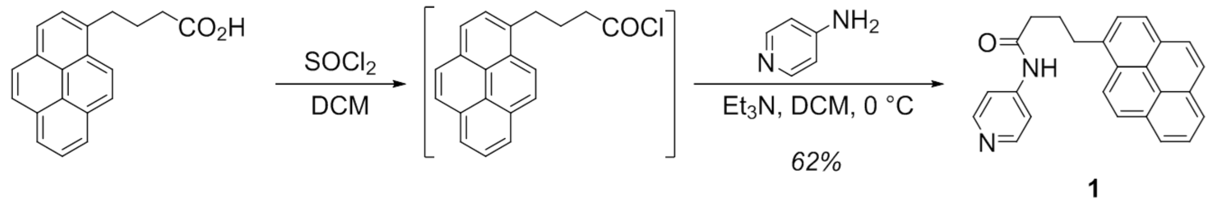

Synthesis and characterization of compound 1. 1-pyrenebutyric acid (300 mg, 1.0 mmol) was suspended in dry DCM (10 mL) under a N_2 atmosphere, before thionyl chloride (2.0 mL, 27.2 mmol) was added dropwise. The solution was stirred for 13 h at room temperature (r.t.) before the solvent was removed under vacuum. The resulting crude was dissolved in dry DCM (5 mL) and added dropwise to a mixture of 4-aminopyridine (147 mg, 1.6 mmol), triethylamine (0.73 mL, 5.2 mmol) in dry DCM (5 mL) at 0 °C. After 3 h stirring at r.t., water was added and the aqueous layer was extracted with ethyl acetate. The combined organic layers were washed with water, brine, dried over sodium sulfate, filtered and concentrated under vacuum. The crude was purified by column chromatography (SiO_2) eluted with a mixture of DCM and methanol (98:2) affording compound **1** as a brown light powder (232 mg, 62%). ^1H NMR ($CDCl_3$, 400 MHz): δ_H (ppm)= 8.44 (d, J = 5.7 Hz, 2H), 8.31 (d, J = 9.2 Hz, 1H), 8.18 (d, J = 7.6 Hz, 2H), 8.12 (m, 2H), 8.03 (s, 2H), 8.00 (m, 1H), 7.88 (d, J = 7.8 Hz, 1H), 7.35 (d, J = 6.2 Hz, 2H), 7.05 (bs, NH, 1H), 3.47 (t, J = 7.2 Hz, 2H), 2.43 (t, J = 6.9 Hz, 2H), 2.33 (m, 2H). ^{13}C NMR ($CDCl_3$, 125 MHz): δ_C (ppm)= 171.5, 150.6, 144.7, 135.2, 131.4, 130.8, 130.1, 128.8, 127.8, 127.6, 127.4, 126.9, 126.0, 125.1, 125.0, 124.9, 124.9, 124.8, 123.2, 113.3, 36.8, 32.3, 26.7. HR-MS (+ESI, m/z): calcd. for $C_{25}H_{21}O_1N_2$ $[M+H]^+$: 365.1648; found, 365.1635. Anal. calcd. for $C_{25}H_{20}N_2O_1 + 0.5H_2O$: C, 80.40; H, 5.67; N, 7.50; found: C, 80.47; H, 5.94; N, 7.03.

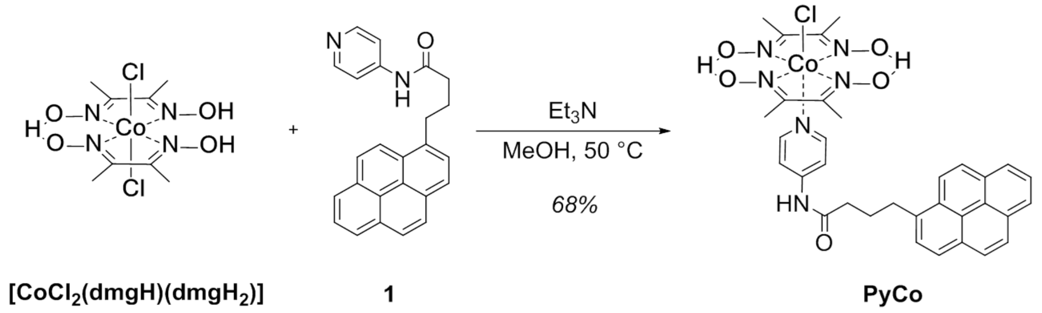

Synthesis and characterization of compound PyCo. Triethylamine (39 µL, 0.28 mmol) was added to a stirred green suspension of $[CoCl_2(dmgH)(dmgH_2)]$ (100 mg, 0.28 mmol) in methanol (7 mL). The color turned quickly from green to brown and the solution was heated at 50 °C for 20 min before **1** (101 mg 0.28 mmol) was added. The solution was then stirred for 2.5 h before cooling down to room temperature. The supernatant was removed and the precipitate was successively washed with ethyl acetate (15 mL), hexane (15 mL) and diethyl ether (15 mL) affording the molecule **PyCo** as a fine brown powder (130 mg, 68%). ^1H NMR (DMSO-d_6, 400 MHz): δ_H (ppm)= 10.67 (s, NH, 1H), 8.38 (d, J = 9.3 Hz, 1H), 8.28 (m, 2H), 8.21 (m, 2H), 8.13, (s, 2H), 8.06 (m, 1H), 7.93 (d, J = 7.1 Hz, 1H), 7.78 (d, J = 7.2 Hz, 2H), 7.53 (d, J = 7.2 Hz, 2H), 3.38 (m, 2H), 2.49 (m, 2H), 2.32 (s, 12H), 2.07 (m, 2H). ^{13}C NMR (DMSO-d_6, 125 MHz): δ_C (ppm)= 173.0, 152.3, 150.4, 150.3, 148.3, 136.2, 130.9, 130.5, 129.4, 128.2, 127.6, 127.5, 127.4, 126.6, 126.2, 125.0, 124.3, 124.2, 123.5, 115.1, 36.0, 32.0, 26.6, 12.6. FT-IR (ATR,

ν/cm^{-1}): 1706, 1594, 1514, 1239, 1212, 1092. HRMS (+ESI, *m/z*): calcd. for $C_{33}H_{35}O_5N_6{}^{35}Cl_1{}^{59}Co_1$ $[M+H]^+$: 689.1684; found, 689.1660. Anal. calcd. for $C_{33}H_{34}Cl_1CoN_6O_5$: C, 57.52; H, 4.97; N, 12.20; Cl, 5.14 found: C, 57.70; H, 4.94; N, 11.89; Cl, 5.04.

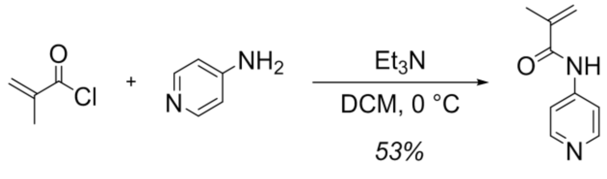

2

Synthesis and characterization of compound 2. 4-Aminopyridine (4.36 g, 46.6 mmol) and triethylamine (26.7 mL, 155 mmol) were placed in a dry round-bottom flask under a N_2 atmosphere and suspended in dry DCM (25 mL). The turbid mixture was cooled to 0 °C before methacryloyl chloride (3.0 mL, 31.1 mmol) was slowly added dropwise and this mixture was stirred overnight at room temperature. The solvent was removed under vacuum and the resulting oil purified by column chromatography (SiO_2) eluted with a mixture of DCM and Et_3N (9:1) affording a colorless oil, which crystalized under high vacuum as a white crystal (2.69 g, 53 %). ^1H NMR ($CDCl_3$, 400 MHz): δ_H (ppm)= 8.52 (d, J = 6.0 Hz, 2H), 7.74 (bs, 1H), 7.53 (d, J = 6.0 Hz, 2H), 5.82 (s, 1H), 5.54 (s, 1H), 2.06 (s, 3H). ^{13}C NMR ($CDCl_3$, 100 MHz): δ_C (ppm)= 167.1, 150.9, 145.0, 140.6, 121.0, 113.8, 18.8. HRMS (+ESI, *m/z*): calcd. for $C_9H_{11}O_1N_2$ $[M+H]^+$: 163.0866; found, 163.0863. Anal. calcd. for $C_9H_{10}N_2O_1$: C, 66.65; H, 6.21; N, 17.27; found: C, 66.19; H, 6.28; N, 16.79.

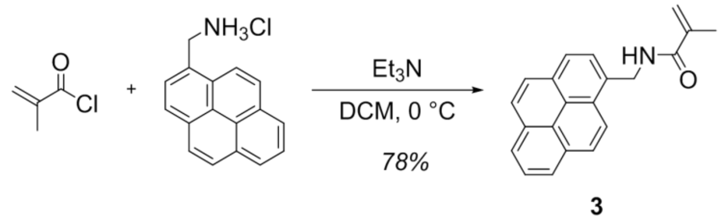

3

Synthesis and characterization of compound 3. Compound **3** was synthesized following the same procedure as for **2**, but using 1-pyrenemethylamine hydrochloride (711 mg, 2.7 mmol) as a starting material instead of 4-aminopyridine. The crude product was purified by column chromatography (SiO_2) using a mixture of DCM and ethyl acetate as eluent (96:4). The product was isolated as a white-yellow fluffy powder (622 mg, 78%). ^1H NMR ($CDCl_3$, 400 MHz): δ_H (ppm)= 8.28 (d, J = 9.2 Hz, 1H), 8.23-8.20 (m, 2H), 8.18-8.15 (m, 2H), 8.07 (m, 2H), 8.04 (d, J = 7.6 Hz, 1H), 7.99 (d, J = 7.6 Hz, 1H), 6.10 (bs, NH, 1H), 5.69 (s, 1H), 5.33 (s, 1H), 5.22 (d, J = 5.2 Hz, 2H), 1.98 (s, 3H). ^{13}C NMR ($CDCl_3$, 100 MHz): δ_C (ppm)= 168.2, 140.0, 131.5, 131.4, 131.1, 130.9, 129.3, 128.5, 127.8, 127.5, 126.3, 125.6, 125.5, 125.2, 124.9, 124.8, 122.9, 119.9, 42.4, 18.9. HRMS (+ESI, *m/z*): calcd. for $C_{21}H_{17}O_1Na_1N_1$ $[M+Na]^+$: 322.1202; found, 322.1190. Anal. calcd. for $C_{21}H_{17}N_1O_1$: C, 84.25; H, 5.72; N, 4.68; found: C, 84.25; H, 5.59; N, 4.82.

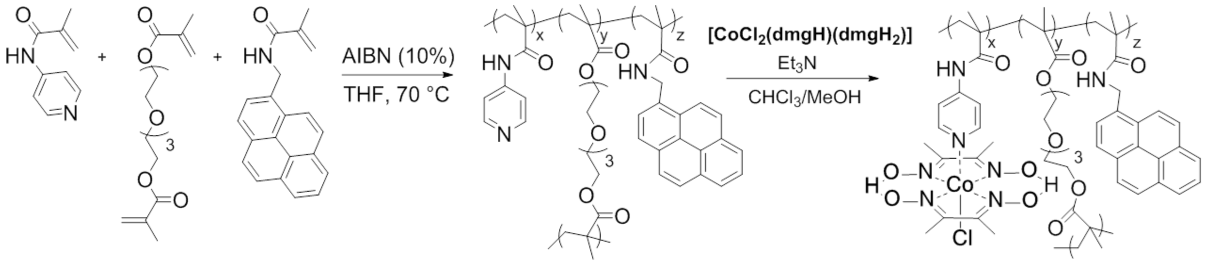

2 4EG 3 pPy pPyCo

Synthesis and characterization of compound pPy. Compound **2** (45 mg, 0.28 mmol), **3** (83 mg, 0.28 mmol) and AIBN (14 mg, 8.3 µmol) were placed in a dry Schlenk flask under a N_2 atmosphere before dry THF (5.5 mL) was added. Tetraethylene glycol dimethacrylate (**4EG**, 85 µL, 0.28 mmol) was then added dropwise and the solution was degassed from O_2 by 3 freeze-pump-thaw cycles. The Schlenk flask was then dipped in a pre-heated oil bath at 70 °C and stirred overnight. After cooling to room temperature, the polymer was precipitated by dropping the light-yellow solution into hexane (50 mL). The solid was filtered off and washed with hexane (20 mL) and ethyl acetate (2 × 10 mL) to remove unreacted molecules and low molecular weight compounds, affording a white solid product, which was then dried overnight under high vacuum (194 mg, 88%). The product was soluble (> 5 mg mL^{-1}) in THF, $CHCl_3$, DMF and DMSO. GPC (PMMA standard, in DMF + 0.1 M LiBr): M_n= 7.2 kDa, $Đ_M$ = 1.26. ^{1}H NMR (DMSO-d_6, 500 MHz): δ_H (ppm)= 9.55 (bs, 1H), 8.8-7.8 (bm, 11H), 7.67 (bs, 2H), 4.87 (bs, 8H), 4.45-2.80 (bm, 22H), 2.3-0.10 (bm, 17H). The molar ratio between x, y & z (see scheme above) was determined by integrating the N-bonded methylene from the pyrene unit, the aromatic protons in the pyridine and in the alkyl proton from [2.3-0] moiety and was found to be approximately 1:1.3:1 (Pyridine:4EG:Pyrene) Anal. found: C, 64.54; H, 6.89; N, 4.38.

Synthesis and characterization of compound pPyCo. [$CoCl_2$(dmgH)(dmgH_2)] (50 mg, 0.14 mmol) was dissolved in a mixture of methanol and chloroform (5 mL, 1:4) and triethylamine (20 µL, 0.15 mmol). In parallel, **pPy** (94 mg) was dissolved in chloroform (5 mL) and added to the brown solution of [$CoCl_2$(dmgH)(dmgH_2)]. The resulting solution was then heated at 45 °C for 4 h. Hexane was added to the solution to complete the precipitation of the polymer. The solid was filtered and washed successively with diethyl ether (10 mL), methanol (10 mL), water (20 mL) and methanol (20 mL) affording the light-brown powder **pPyCo**, which was dried under high vacuum at room temperature overnight (100 mg, 69%). The product was soluble (> 5 mg mL^{-1}) in DMF and DMSO. GPC (PMMA standard, in DMF + 0.1 M LiBr): M_n= 8.3 kDa, $Đ_M$ = 1.29. ^{1}H NMR (DMSO-d_6, 500 MHz): δ (ppm)= 18.44 (bs, 2H), 9.79 (bs, 1H), 8.63-7.20 (bm, 13H), 4.88 (bs, 2H), 4.35-2.71 (bm, 22H), 2.39-0.1 (bm, 29H). FT-IR (ATR, ν/cm^{-1}): 1721 (s), 1240, 1131 (s), 1093 (s). Anal. found: C, 54.05; H, 6.16; N, 7.37; Cl, 3.21. Elemental analysis and NMR experiments revealed that the molar ratio between x, y & z (see scheme above) has not been changed by the reaction, indicating a quantitative complexation of the pyridine ligands.

Fabrication of the GC/MWCNT electrodes. GC/MWCNT electrodes were obtained by drop-coating 20 µL of a 5 mg mL^{-1} dispersion of MWCNTs in *N*-methylpyrrolidone onto the GC disk electrode (ø = 3 mm). After drying under vacuum, a 5 µm thick homogeneous MWCNT film was obtained as reported previously.[B. Reuillard, S. Gentil, M. Carrière, A. L. Goff, S. Cosnier, *Chem. Sci*. **2015**, *6*, 5139–5143]. For immobilization of **PyCo** and **pPyCo** the GC/MWCNT electrodes were then incubated

for 30 min in a 2.5 mM solution of **PyCo** (GC/MWCNT-**PyCo**) or **pPyCo** (GC/MWCNT-**pPyCo**) in DMF and finally rinsed with DMF and H_2O.

Fabrication of the MWCNT buckypapers. The buckypaper electrodes were prepared by dispersing MWCNTs (30 mg) in DMF (150 mL) for 30 min in an ultrasonic bath. BP-**PyCo** (30 mg), BP-**pPyCo** (30 mg) or BP-**pPy** (30 mg) was subsequently added to the black dispersion and the mixture was allowed to stir overnight at room temperature. The dispersion was then filtered through a polytetrafluoroethylene (PTFE) membrane (0.45 µm pore size), washed with acetone (30 mL) and water (50 mL). The as-formed buckypaper was then carefully removed from the membrane and allowed to dry. The bare buckypaper was obtained following the same procedure but without adding any catalyst.

Electrochemistry. Cyclic voltammetry and chronoamperometry measurements were performed with an Ivium CompactStat or an IviumStat potentiostat. A three-electrode configuration was employed: a glassy carbon electrode (ø = 3 mm) or the buckypaper (geometric surface area = 1 cm^2) were set as working electrode, a Pt mesh was used as counter electrode and an Ag/AgCl electrode (satd. KCl solution) as reference electrode. All measurements were performed in aqueous media in phosphate buffer (0.1 M) at pH 6.5 and room temperature.

Controlled-potential electrolysis (CPE). CPE of BP, BP-**PyCo** and BP-**pPyCo** was carried out with stirring at E_{appl} = –0.7 V *vs.* SHE in an aqueous pH 6.5 phosphate buffer solution (0.1 M). A N_2 (containing 2% CH_4 as internal standard for GC measurements) or air atmosphere was used and all measurements were recorded at room temperature. An airtight two-compartment three-necked cell with a three-electrode configuration was employed: the buckypaper working electrode (1 cm^2) and the Ag/AgCl reference electrode were separated from the Pt mesh counter electrode by a Nafion membrane to avoid the diffusion of O_2 produced at the counter electrode during the electrolysis. The amount of hydrogen produced was quantified by gas chromatography (GC; see below). The faradaic efficiencies were calculated using the following equation:

$$Faradic\ yield = 100 \times \frac{(2\mathrm{F}[H_2])}{\mathrm{Q}}$$

Where F is the Faraday constant (C mol^{-1}), [H_2] (mol) is the amount of hydrogen measured in the headspace and Q (C) is the charged passed during electrolysis.

Headspace H_2 gas analysis. The amount of gaseous H_2 was detected and quantified by headspace gas analysis using an Agilent 7890A Series GC equipped with a 5 Å molecular sieve column (N_2 carrier gas at a flow rate of approximately 3 mL min^{-1}). The GC oven holding the columns was kept isothermal at 45 ^{o}C, and a thermal conductivity detector was employed. Aliquots (40 µL) of the headspace gas were removed for GC analysis after 0, 0.083, 0.25, 0.5, 1, 2, 3, 4, 5, 6, 7, 8, 10, 24 and 25 h. The corrected amount of H_2 produced with BP-**PyCo** and BP-**pPyCo** were calculated by subtracting the values obtained with the corresponding bare BP and BP-**pPy** respectively. Analytical measurements were performed in triplicate and errors are given as mean standard deviation.

Supporting Figures

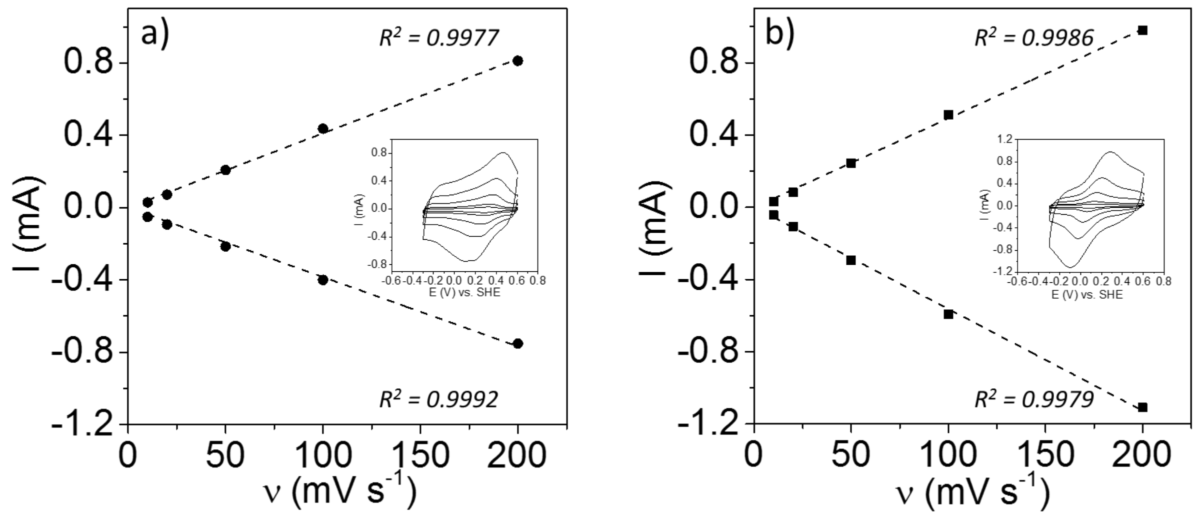

Figure S1. Peak current *vs.* scan rate for both reduction and oxidation waves of a) **PyCo** and b) **pPyCo**, immobilized on GC/MWCNT electrodes. Insets: CV scans at different scan rates (10, 20, 50, 100 and 200 mV s^{-1}) performed in phosphate buffer (0.1 M, pH 6.5).

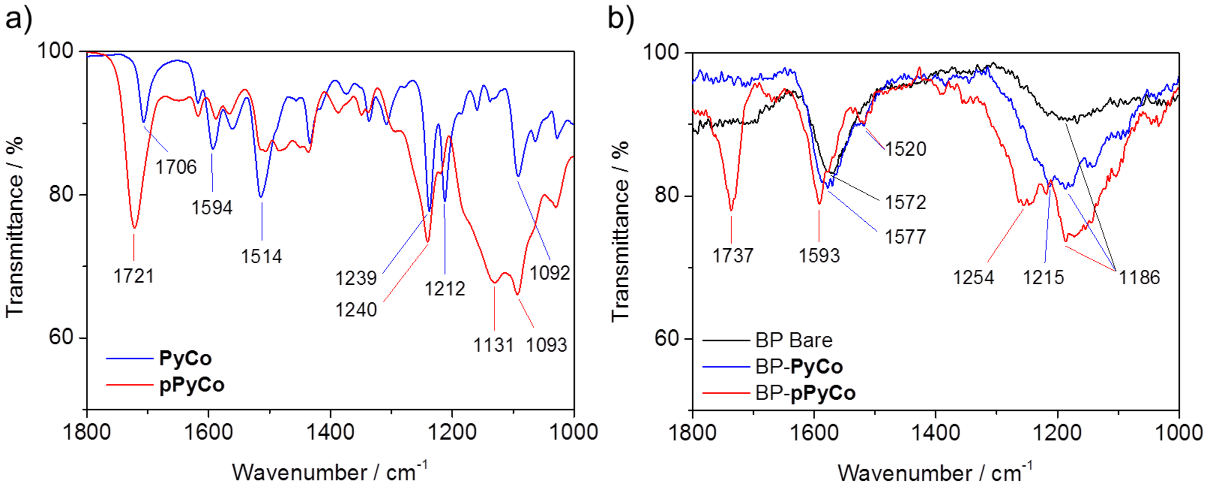

Figure S2. Attenuated total reflectance FT-IR spectra of a) **PyCo** (blue) & **pPyCo** (red), and b) bare BP (black), BP-**pPyCo** (red) & BP-**PyCo** (blue trace).

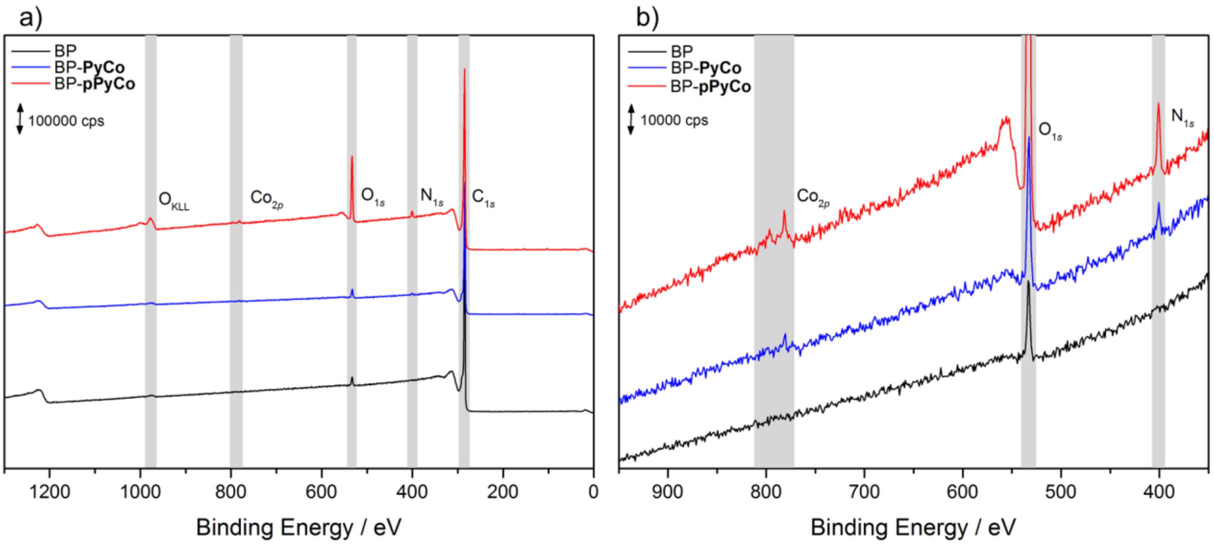

Figure S3. XPS analysis a) survey and b) enlargement of the Co_{2p}, O_{1s} and N_{1s} regions of the pristine BP (black), BP-**PyCo** (blue) and BP-**pPyCo** (red trace).

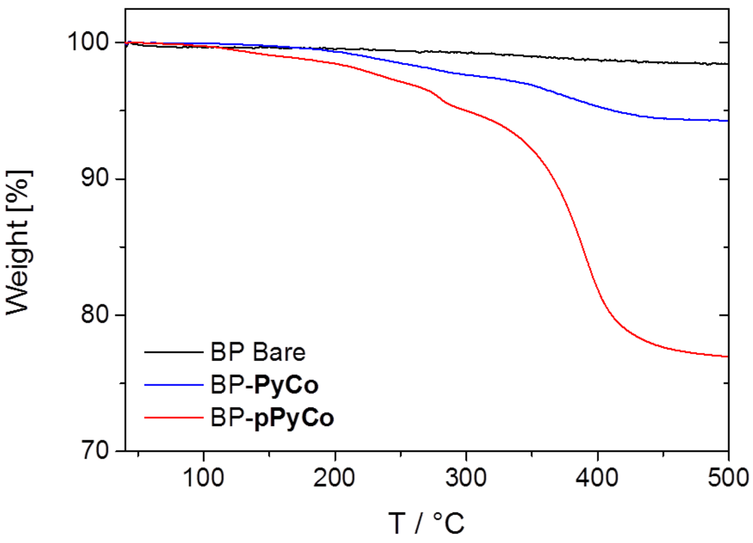

Figure S4. Thermogravimetric analysis traces (under N_2, with a 10 °C min^{-1} gradient of temperature) of bare BP (black), BP-**PyCo** (blue) and BP-**pPyCo** (red trace).

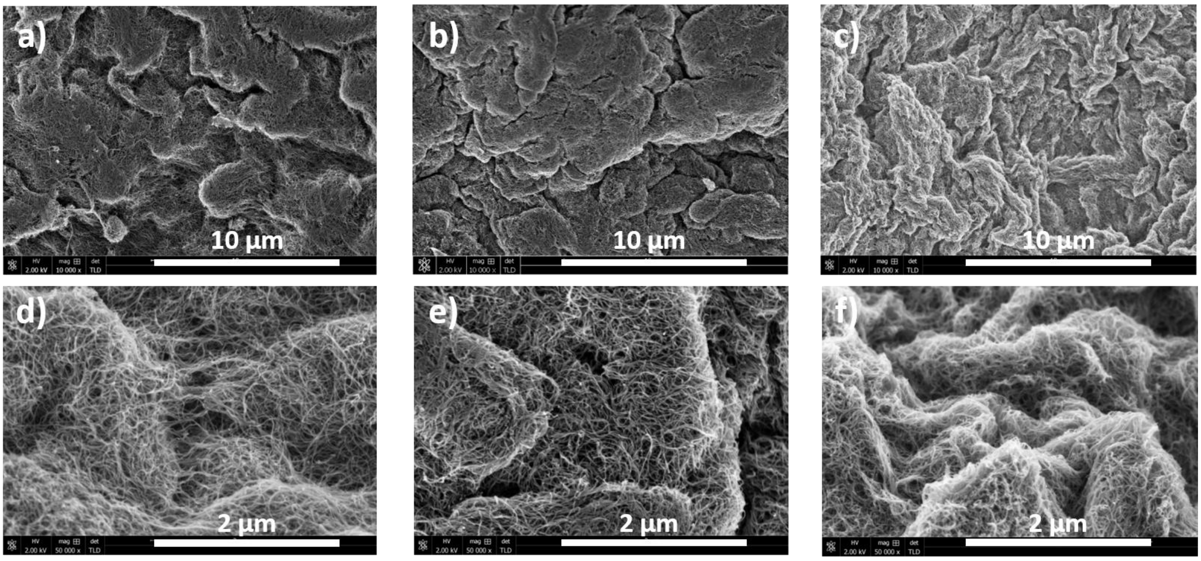

Figure S5. SEM images of bare BP (a & d), BP-**PyCo** (b & e) and BP-**pPyCo** (c & f).

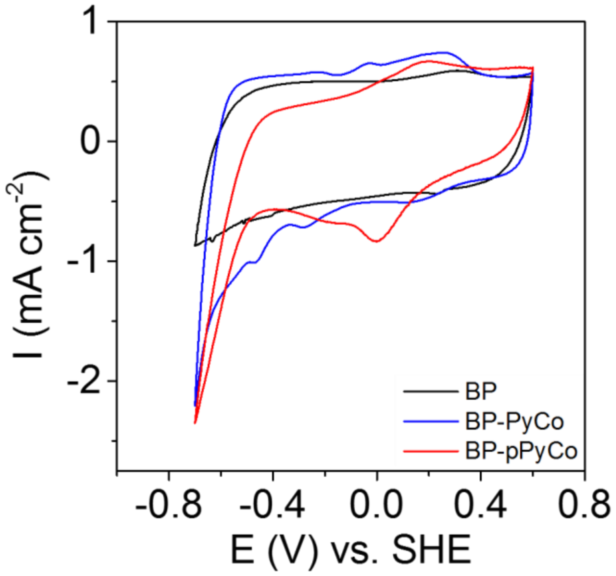

Figure S6. CV of the different BPs with a larger potential window showing the catalytic reduction of protons, recorded in phosphate buffer (0.1 M, pH 6.5) with a scan rate of $v = 5$ mV s^{-1} under an inert atmosphere.

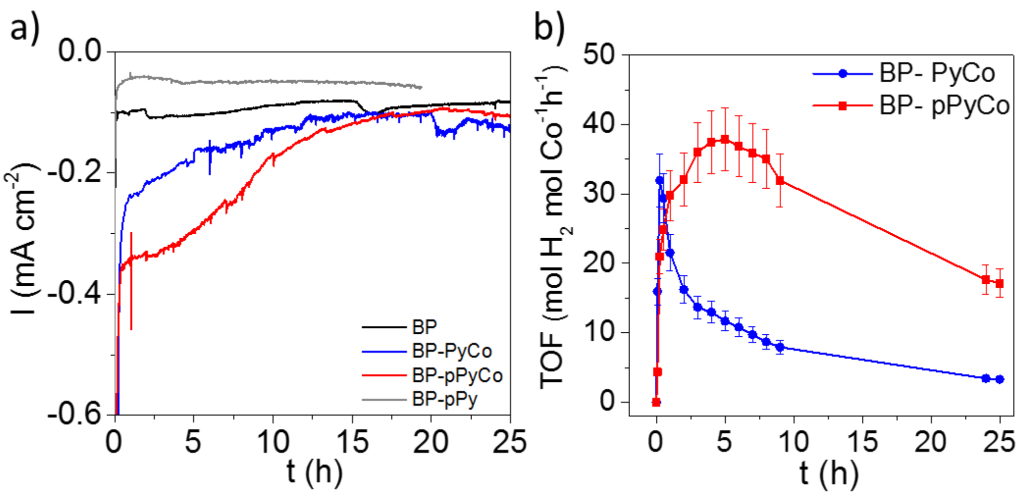

Figure S7. a) Current density and b) turnover frequency evolution over time during controlled-potential electrolysis experiments conducted on bare BP (black), BP-**pPy** (grey, conducted for 20 h) BP-**PyCo** (blue) and BP-**pPyCo** (red curves), at E_{appl} = −0.7 V *vs.* SHE in phosphate buffer (0.1 M, pH 6.5).

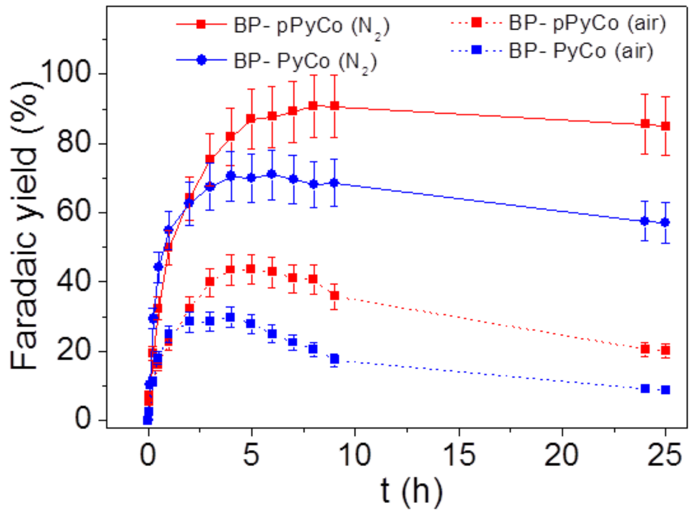

Figure S8. Faradaic yield for H_2 evolution during CPE experiments conducted on BP-**pPyCo** (red curves) and BP-**PyCo** (blue curves) in the presence of oxygen (dotted line) or under an inert atmosphere (solid line) at E_{appl} = −0.7 V *vs.* SHE in phosphate buffer (0.1 M, pH 6.5).

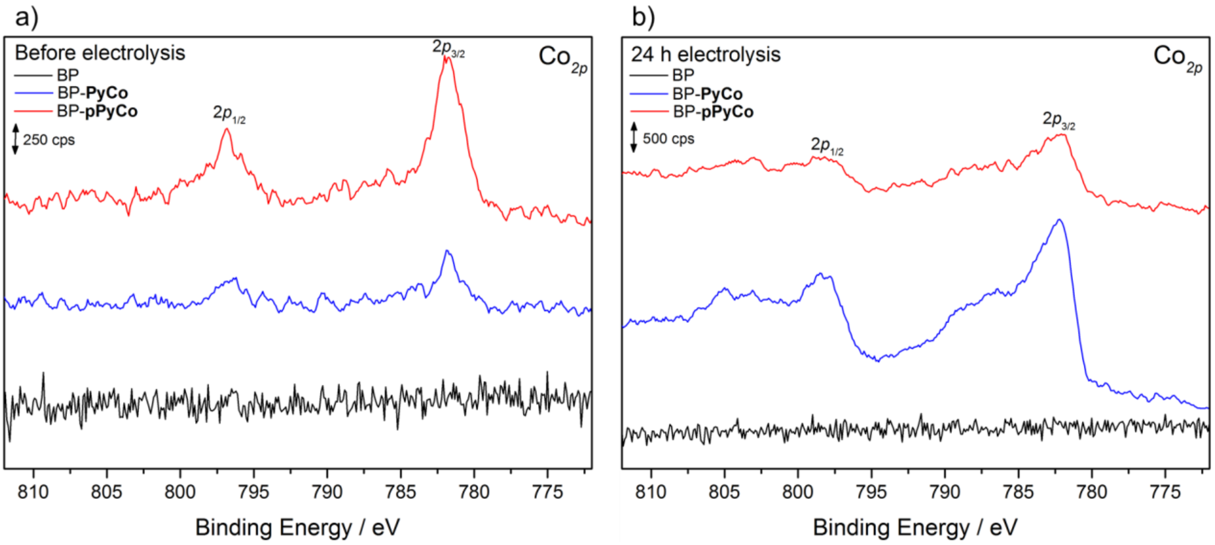

Figure S9. XPS analysis of the Co_{2p} region of the pristine BP (black), BP-**PyCo** (blue) and BP-**pPyCo** (red trace) a) before and b) after CPE (24 h).

End of Supporting Information
